# *N*-(3,5-Dimethyl­phen­yl)-2,4-dimethyl­benzene­sulfonamide

**DOI:** 10.1107/S1600536809050740

**Published:** 2009-11-28

**Authors:** B. Thimme Gowda, Sabine Foro, P. G. Nirmala, Hartmut Fuess

**Affiliations:** a Department of Chemistry, Mangalore University, Mangalagangotri 574 199, Mangalore, India; b Institute of Materials Science, Darmstadt University of Technology, Petersenstrasse 23, D-64287 Darmstadt, Germany

## Abstract

In the title compound, C_16_H_19_NO_2_S, the mol­ecule is twisted about the S—N bond, the C—S(O_2_)—N(H)—C torsion angle being 53.9 (2)°. The dihedral angle between the two benzene rings is 82.1 (1)°. The crystal structure features inversion-related dimers linked by N—H⋯O hydrogen bonds.

## Related literature

For the preparation of the title compound, see: Savitha & Gowda (2006 ▶). For our work on the effect of substituents on the structures of *N*-(ar­yl)aryl­sulfonamides, see: Gowda *et al.* (2009*a* ▶,*b* ▶); Nirmala *et al.* (2009 ▶). For related structures, see: Gelbrich *et al.* (2007 ▶); Perlovich *et al.* (2006 ▶).
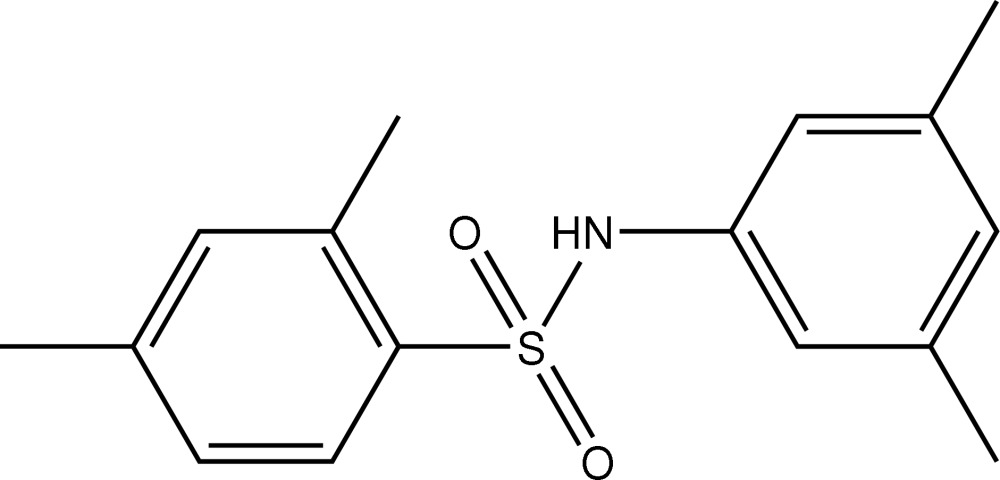


## Experimental

### 

#### Crystal data

C_16_H_19_NO_2_S
*M* *_r_* = 289.38
Monoclinic, 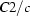

*a* = 23.490 (2) Å
*b* = 8.1528 (6) Å
*c* = 16.544 (1) Å
β = 102.690 (8)°
*V* = 3090.9 (4) Å^3^
*Z* = 8
Mo *K*α radiation
μ = 0.21 mm^−1^
*T* = 299 K
0.40 × 0.20 × 0.12 mm

#### Data collection

Oxford Diffraction Xcalibur diffractometer with Sapphire CCD detector
Absorption correction: multi-scan (*CrysAlis RED*; Oxford Diffraction, 2009 ▶) *T*_min_ = 0.921, *T*_max_ = 0.975
6232 measured reflections
2751 independent reflections
2009 reflections with *I* > 2σ(*I*)
*R*_int_ = 0.018

#### Refinement

*R*[*F* ^2^ > 2σ(*F* ^2^)] = 0.044
*wR*(*F* ^2^) = 0.118
*S* = 1.01
2751 reflections
188 parameters
1 restraint
H atoms treated by a mixture of independent and constrained refinement
Δρ_max_ = 0.19 e Å^−3^
Δρ_min_ = −0.28 e Å^−3^

### 

Data collection: *CrysAlis CCD* (Oxford Diffraction, 2009 ▶); cell refinement: *CrysAlis RED* (Oxford Diffraction, 2009 ▶); data reduction: *CrysAlis RED*; program(s) used to solve structure: *SHELXS97* (Sheldrick, 2008 ▶); program(s) used to refine structure: *SHELXL97* (Sheldrick, 2008 ▶); molecular graphics: *PLATON* (Spek, 2009 ▶); software used to prepare material for publication: *SHELXL97*.

## Supplementary Material

Crystal structure: contains datablocks I, global. DOI: 10.1107/S1600536809050740/tk2587sup1.cif

Structure factors: contains datablocks I. DOI: 10.1107/S1600536809050740/tk2587Isup2.hkl

Additional supplementary materials:  crystallographic information; 3D view; checkCIF report

## Figures and Tables

**Table 1:** Hydrogen-bond geometry (Å, °)

*D*—H⋯*A*	*D*—H	H⋯*A*	*D*⋯*A*	*D*—H⋯*A*
N1—H1*N*⋯O1^i^	0.84 (2)	2.10 (2)	2.945 (3)	176 (3)

Symmetry code: (i) 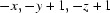.

**Fig. 1.**
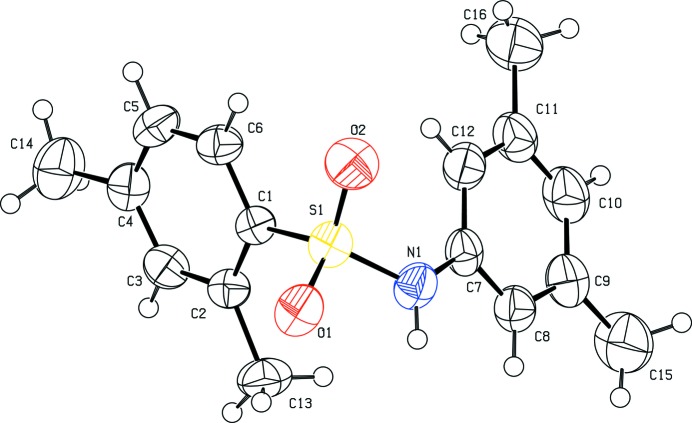
Molecular structure of (I), showing the atom labelling scheme. The displacement ellipsoids are drawn at the 50% probability level. The H atoms are represented as small spheres of arbitrary radii.

## supplementary crystallographic information

### Comment

As part of a study of substituent effects on the structures of *N*-(aryl)arylsulfonamides (Gowda *et al.*, 2009*a,b*; Nirmala *et al.*, 2009), in the present work, the structure of 2,4-dimethyl-*N*-(3,5-dimethylphenyl)benzenesulfonamide (I) has been determined (Fig. 1). The molecule is bent at the S–O bond atom with the C1—SO_2_—NH—C7 torsion angle being 53.9 (2)°, compared to the values of 46.1 (3)° and 47.7 (3)° in the two independent molecules of 2,4-dimethyl-*N*-(3,5-dimethylphenyl)benzenesulfonamide (II) (Gowda *et al.*, 2009*a*), 67.9 (2)° in *N*-(3,5-dimethylphenyl)benzenesulfonamide (III) (Nirmala *et al.*, 2009) and -69.7 (2)° in 2,4-dimethyl-*N*-(3,4-dichlorophenyl)benzenesulfonamide (IV) (Gowda *et al.*, 2009*b*).

The two benzene rings in (I) are tilted relative to each other by 82.1 (1)°, compared to the values of 67.5 (1)° (molecule 1) and 72.9 (1)° (molecule 2) in the two independent molecules of (II), 54.6 (1)° in (III) and 82.4 (1)° in (IV). The other bond parameters in (I) are similar to those observed in (II), (III), (IV) and other aryl sulfonamides (Perlovich *et al.*, 2006; Gelbrich *et al.*, 2007). The crystal packing of molecules in (I) is through pairs of N—H···O(S) hydrogen bonds (Table 1).

### Experimental

The solution of *m*-xylene (10 cc) in chloroform (40 cc) was treated drop-wise with chlorosulfonic acid (25 ml) at 273 K. After the initial evolution of hydrogen chloride subsided, the reaction mixture was brought to room temperature and poured into crushed ice in a beaker. The chloroform layer was separated, washed with cold water and allowed to evaporate slowly. The residual 2,4-dimethylbenzenesulfonylchloride was treated with a stoichiometric ratio of 3,4-dimethylaniline and boiled for ten minutes. The reaction mixture was then cooled to room temperature and added to ice-cold water (100 ml). The resultant solid, 2,4-dimethyl-*N*- (3,5-dimethylphenyl)benzenesulfonamide, was filtered under suction and washed thoroughly with cold water. It was then recrystallized to constant melting point from dilute ethanol. The purity of the compound was checked and characterized by recording its infrared and NMR spectra (Savitha & Gowda, 2006). The single crystals used in X-ray diffraction studies were grown in ethanolic solution by slow evaporation at room temperature.

### Refinement

The H atom of the NH group was located in a difference map and refined with the distance restraint N—H = 0.86 (2) Å, and with U_iso_(H) = 1.2*U*_eq_(C). The C atoms were included in the riding model approximation with C—H = 0.93–0.96 Å, and with U_iso_(H) = 1.2*U*_eq_(C).

### Crystal data

**Table:**

C_16_H_19_NO_2_S	*F*(000) = 1232
*M**_r_* = 289.38	*D*_x_ = 1.244 Mg m^−^^3^
Monoclinic, *C*2/*c*	Mo *K*α radiation, λ = 0.71073 Å
Hall symbol: -C 2yc	Cell parameters from 2194 reflections
*a* = 23.490 (2) Å	θ = 2.5–27.9°
*b* = 8.1528 (6) Å	µ = 0.21 mm^−^^1^
*c* = 16.544 (1) Å	*T* = 299 K
β = 102.690 (8)°	Prism, colourless
*V* = 3090.9 (4) Å^3^	0.40 × 0.20 × 0.12 mm
*Z* = 8	

### Data collection

**Table:**

Oxford Diffraction Xcalibur diffractometer with Sapphire CCD detector	2751 independent reflections
Radiation source: fine-focus sealed tube	2009 reflections with *I* > 2σ(*I*)
graphite	*R*_int_ = 0.018
Rotation method data acquisition using ω and φ scans.	θ_max_ = 25.3°, θ_min_ = 2.7°
Absorption correction: multi-scan (*CrysAlis RED*; Oxford Diffraction, 2009)	*h* = −28→17
*T*_min_ = 0.921, *T*_max_ = 0.975	*k* = −9→9
6232 measured reflections	*l* = −19→19

### Refinement

**Table:**

Refinement on *F*^2^	Primary atom site location: structure-invariant direct methods
Least-squares matrix: full	Secondary atom site location: difference Fourier map
*R*[*F*^2^ > 2σ(*F*^2^)] = 0.044	Hydrogen site location: inferred from neighbouring sites
*wR*(*F*^2^) = 0.118	H atoms treated by a mixture of independent and constrained refinement
*S* = 1.01	*w* = 1/[σ^2^(*F*_o_^2^) + (0.0512*P*)^2^ + 3.0162*P*] where *P* = (*F*_o_^2^ + 2*F*_c_^2^)/3
2751 reflections	(Δ/σ)_max_ = 0.004
188 parameters	Δρ_max_ = 0.19 e Å^−^^3^
1 restraint	Δρ_min_ = −0.28 e Å^−^^3^

### Special details

**Table:**

Geometry. All e.s.d.'s (except the e.s.d. in the dihedral angle between two l.s. planes) are estimated using the full covariance matrix. The cell e.s.d.'s are taken into account individually in the estimation of e.s.d.'s in distances, angles and torsion angles; correlations between e.s.d.'s in cell parameters are only used when they are defined by crystal symmetry. An approximate (isotropic) treatment of cell e.s.d.'s is used for estimating e.s.d.'s involving l.s. planes.
Refinement. Refinement of *F*^2^ against ALL reflections. The weighted *R*-factor *wR* and goodness of fit *S* are based on *F*^2^, conventional *R*-factors *R* are based on *F*, with *F* set to zero for negative *F*^2^. The threshold expression of *F*^2^ > σ(*F*^2^) is used only for calculating *R*-factors(gt) *etc*. and is not relevant to the choice of reflections for refinement. *R*-factors based on *F*^2^ are statistically about twice as large as those based on *F*, and *R*- factors based on ALL data will be even larger.

### Fractional atomic coordinates and isotropic or equivalent isotropic displacement parameters (Å^2^)

**Table:**

	*x*	*y*	*z*	*U*_iso_*/*U*_eq_	
S1	0.05610 (3)	0.54362 (8)	0.39886 (4)	0.0472 (2)	
O1	−0.00491 (7)	0.5510 (2)	0.40012 (10)	0.0581 (5)	
O2	0.08128 (8)	0.6784 (2)	0.36408 (12)	0.0655 (5)	
N1	0.08841 (9)	0.5250 (3)	0.49593 (13)	0.0519 (5)	
H1N	0.0648 (10)	0.498 (3)	0.5253 (15)	0.062*	
C1	0.07002 (9)	0.3654 (3)	0.34572 (13)	0.0382 (5)	
C2	0.05851 (9)	0.2067 (3)	0.37077 (14)	0.0421 (5)	
C3	0.06794 (10)	0.0785 (3)	0.32105 (16)	0.0492 (6)	
H3	0.0611	−0.0277	0.3372	0.059*	
C4	0.08716 (10)	0.0999 (3)	0.24799 (16)	0.0498 (6)	
C5	0.09849 (10)	0.2566 (3)	0.22560 (15)	0.0525 (6)	
H5	0.1119	0.2739	0.1774	0.063*	
C6	0.09019 (10)	0.3886 (3)	0.27382 (15)	0.0480 (6)	
H6	0.0982	0.4940	0.2580	0.058*	
C7	0.14835 (10)	0.4895 (3)	0.52826 (15)	0.0458 (6)	
C8	0.16296 (10)	0.4248 (3)	0.60749 (15)	0.0508 (6)	
H8	0.1337	0.4019	0.6357	0.061*	
C9	0.22056 (11)	0.3939 (3)	0.64514 (17)	0.0606 (7)	
C10	0.26309 (12)	0.4250 (4)	0.60070 (19)	0.0675 (8)	
H10	0.3019	0.4029	0.6252	0.081*	
C11	0.24971 (11)	0.4873 (4)	0.52134 (18)	0.0612 (8)	
C12	0.19150 (11)	0.5218 (3)	0.48498 (17)	0.0564 (7)	
H12	0.1817	0.5663	0.4320	0.068*	
C13	0.03632 (13)	0.1703 (4)	0.44804 (16)	0.0636 (7)	
H13A	0.0022	0.2352	0.4479	0.076*	
H13B	0.0660	0.1966	0.4961	0.076*	
H13C	0.0266	0.0561	0.4491	0.076*	
C14	0.09508 (14)	−0.0463 (4)	0.1958 (2)	0.0796 (9)	
H14A	0.0580	−0.0979	0.1752	0.096*	
H14B	0.1212	−0.1231	0.2289	0.096*	
H14C	0.1111	−0.0109	0.1501	0.096*	
C15	0.23625 (14)	0.3267 (5)	0.7320 (2)	0.0931 (11)	
H15A	0.2522	0.2184	0.7310	0.112*	
H15B	0.2019	0.3220	0.7544	0.112*	
H15C	0.2647	0.3968	0.7659	0.112*	
C16	0.29662 (13)	0.5197 (5)	0.4739 (2)	0.0881 (11)	
H16A	0.3020	0.4238	0.4428	0.106*	
H16B	0.3325	0.5457	0.5121	0.106*	
H16C	0.2852	0.6102	0.4368	0.106*	

### Atomic displacement parameters (Å^2^)

**Table:**

	*U*^11^	*U*^22^	*U*^33^	*U*^12^	*U*^13^	*U*^23^
S1	0.0477 (4)	0.0440 (4)	0.0490 (4)	0.0102 (3)	0.0089 (3)	0.0005 (3)
O1	0.0450 (9)	0.0733 (12)	0.0548 (10)	0.0213 (9)	0.0082 (8)	−0.0019 (10)
O2	0.0843 (13)	0.0390 (10)	0.0738 (13)	0.0028 (9)	0.0189 (10)	0.0074 (10)
N1	0.0434 (12)	0.0667 (14)	0.0455 (12)	0.0089 (10)	0.0098 (9)	−0.0063 (11)
C1	0.0364 (11)	0.0397 (13)	0.0386 (12)	0.0039 (9)	0.0083 (9)	0.0025 (10)
C2	0.0403 (13)	0.0429 (13)	0.0435 (12)	0.0022 (10)	0.0103 (10)	0.0042 (11)
C3	0.0502 (14)	0.0397 (13)	0.0576 (15)	0.0004 (11)	0.0114 (12)	0.0040 (12)
C4	0.0441 (13)	0.0535 (16)	0.0507 (14)	0.0080 (11)	0.0078 (11)	−0.0066 (13)
C5	0.0549 (15)	0.0656 (18)	0.0405 (13)	0.0069 (13)	0.0177 (11)	0.0018 (13)
C6	0.0504 (14)	0.0478 (14)	0.0471 (14)	0.0012 (11)	0.0134 (11)	0.0089 (12)
C7	0.0405 (13)	0.0456 (14)	0.0498 (14)	0.0034 (10)	0.0065 (10)	−0.0145 (12)
C8	0.0473 (14)	0.0528 (15)	0.0512 (15)	0.0002 (11)	0.0086 (11)	−0.0078 (13)
C9	0.0523 (16)	0.0623 (17)	0.0615 (17)	−0.0007 (13)	0.0002 (13)	−0.0054 (15)
C10	0.0425 (15)	0.080 (2)	0.074 (2)	0.0033 (14)	−0.0010 (13)	−0.0087 (17)
C11	0.0429 (14)	0.0707 (19)	0.0708 (19)	−0.0047 (13)	0.0140 (13)	−0.0191 (16)
C12	0.0518 (15)	0.0667 (18)	0.0498 (14)	−0.0008 (13)	0.0095 (12)	−0.0092 (14)
C13	0.0804 (19)	0.0584 (17)	0.0598 (17)	−0.0053 (14)	0.0324 (14)	0.0097 (15)
C14	0.089 (2)	0.075 (2)	0.076 (2)	0.0131 (18)	0.0195 (17)	−0.0212 (18)
C15	0.069 (2)	0.116 (3)	0.083 (2)	−0.0011 (19)	−0.0087 (16)	0.024 (2)
C16	0.0550 (18)	0.120 (3)	0.094 (2)	−0.0072 (18)	0.0268 (16)	−0.016 (2)

### Geometric parameters (Å, °)

**Table:**

S1—O2	1.4276 (18)	C8—H8	0.9300
S1—O1	1.4392 (17)	C9—C10	1.388 (4)
S1—N1	1.626 (2)	C9—C15	1.506 (4)
S1—C1	1.766 (2)	C10—C11	1.378 (4)
N1—C7	1.422 (3)	C10—H10	0.9300
N1—H1N	0.844 (16)	C11—C12	1.396 (3)
C1—C6	1.386 (3)	C11—C16	1.510 (4)
C1—C2	1.403 (3)	C12—H12	0.9300
C2—C3	1.378 (3)	C13—H13A	0.9600
C2—C13	1.512 (3)	C13—H13B	0.9600
C3—C4	1.391 (3)	C13—H13C	0.9600
C3—H3	0.9300	C14—H14A	0.9600
C4—C5	1.373 (4)	C14—H14B	0.9600
C4—C14	1.507 (4)	C14—H14C	0.9600
C5—C6	1.379 (3)	C15—H15A	0.9600
C5—H5	0.9300	C15—H15B	0.9600
C6—H6	0.9300	C15—H15C	0.9600
C7—C8	1.384 (3)	C16—H16A	0.9600
C7—C12	1.389 (3)	C16—H16B	0.9600
C8—C9	1.383 (3)	C16—H16C	0.9600
			
O2—S1—O1	118.40 (11)	C10—C9—C15	121.2 (3)
O2—S1—N1	108.98 (12)	C11—C10—C9	122.1 (2)
O1—S1—N1	103.88 (11)	C11—C10—H10	118.9
O2—S1—C1	107.12 (11)	C9—C10—H10	118.9
O1—S1—C1	109.61 (10)	C10—C11—C12	118.8 (2)
N1—S1—C1	108.52 (11)	C10—C11—C16	121.3 (3)
C7—N1—S1	126.83 (17)	C12—C11—C16	119.9 (3)
C7—N1—H1N	117.0 (19)	C7—C12—C11	119.8 (3)
S1—N1—H1N	111.7 (19)	C7—C12—H12	120.1
C6—C1—C2	120.2 (2)	C11—C12—H12	120.1
C6—C1—S1	116.78 (18)	C2—C13—H13A	109.5
C2—C1—S1	122.89 (17)	C2—C13—H13B	109.5
C3—C2—C1	117.2 (2)	H13A—C13—H13B	109.5
C3—C2—C13	119.1 (2)	C2—C13—H13C	109.5
C1—C2—C13	123.7 (2)	H13A—C13—H13C	109.5
C2—C3—C4	123.3 (2)	H13B—C13—H13C	109.5
C2—C3—H3	118.3	C4—C14—H14A	109.5
C4—C3—H3	118.3	C4—C14—H14B	109.5
C5—C4—C3	118.1 (2)	H14A—C14—H14B	109.5
C5—C4—C14	121.7 (2)	C4—C14—H14C	109.5
C3—C4—C14	120.2 (3)	H14A—C14—H14C	109.5
C4—C5—C6	120.7 (2)	H14B—C14—H14C	109.5
C4—C5—H5	119.7	C9—C15—H15A	109.5
C6—C5—H5	119.7	C9—C15—H15B	109.5
C5—C6—C1	120.6 (2)	H15A—C15—H15B	109.5
C5—C6—H6	119.7	C9—C15—H15C	109.5
C1—C6—H6	119.7	H15A—C15—H15C	109.5
C8—C7—C12	120.2 (2)	H15B—C15—H15C	109.5
C8—C7—N1	116.9 (2)	C11—C16—H16A	109.5
C12—C7—N1	122.8 (2)	C11—C16—H16B	109.5
C9—C8—C7	120.7 (2)	H16A—C16—H16B	109.5
C9—C8—H8	119.7	C11—C16—H16C	109.5
C7—C8—H8	119.7	H16A—C16—H16C	109.5
C8—C9—C10	118.4 (3)	H16B—C16—H16C	109.5
C8—C9—C15	120.4 (3)		
			
O2—S1—N1—C7	−62.4 (2)	C14—C4—C5—C6	179.1 (2)
O1—S1—N1—C7	170.5 (2)	C4—C5—C6—C1	−0.2 (4)
C1—S1—N1—C7	53.9 (2)	C2—C1—C6—C5	0.8 (3)
O2—S1—C1—C6	−12.4 (2)	S1—C1—C6—C5	−175.71 (18)
O1—S1—C1—C6	117.20 (18)	S1—N1—C7—C8	−158.6 (2)
N1—S1—C1—C6	−129.97 (18)	S1—N1—C7—C12	23.5 (4)
O2—S1—C1—C2	171.16 (18)	C12—C7—C8—C9	1.0 (4)
O1—S1—C1—C2	−59.2 (2)	N1—C7—C8—C9	−177.0 (2)
N1—S1—C1—C2	53.6 (2)	C7—C8—C9—C10	−1.8 (4)
C6—C1—C2—C3	−0.2 (3)	C7—C8—C9—C15	178.8 (3)
S1—C1—C2—C3	176.07 (17)	C8—C9—C10—C11	1.0 (4)
C6—C1—C2—C13	−179.8 (2)	C15—C9—C10—C11	−179.6 (3)
S1—C1—C2—C13	−3.6 (3)	C9—C10—C11—C12	0.6 (4)
C1—C2—C3—C4	−1.0 (3)	C9—C10—C11—C16	−179.8 (3)
C13—C2—C3—C4	178.7 (2)	C8—C7—C12—C11	0.7 (4)
C2—C3—C4—C5	1.5 (4)	N1—C7—C12—C11	178.5 (2)
C2—C3—C4—C14	−178.5 (2)	C10—C11—C12—C7	−1.5 (4)
C3—C4—C5—C6	−0.9 (4)	C16—C11—C12—C7	179.0 (3)

### Hydrogen-bond geometry (Å, °)

**Table:**

*D*—H···*A*	*D*—H	H···*A*	*D*···*A*	*D*—H···*A*
N1—H1N···O1^i^	0.84 (2)	2.10 (2)	2.945 (3)	176 (3)

Symmetry codes: (i) −*x*, −*y*+1, −*z*+1.
